# Sensory and vascular improvement following re-release of amniotic band syndrome and reconstruction with free anterolateral thigh flap in late childhood

**DOI:** 10.1016/j.jpra.2025.10.023

**Published:** 2025-11-01

**Authors:** Mardhiah Jeffrey, Liu Yi, Ahmad Sukari Halim, Abdul Razak Sulaiman, Bazli Md Yusoff

**Affiliations:** a Reconstructive Sciences Unit, School of Medical Sciences, Universiti Sains Malaysia, Kelantan, Malaysia; b Hospital Pakar Universiti Sains Malaysia, Kelantan, Malaysia; c Department of Orthopaedics, School of Medical Science, Universiti Sains Malaysia, Kelantan, Malaysia; d Department of Radiology, School of Medical Science, Universiti Sains Malaysia, Kelantan, Malaysia

**Keywords:** Amniotic band syndrome, Sensory, Vascular changes, Anterolateral thigh flap

## Abstract

We report a 9-year-old boy with a residual amniotic band over the middle of right leg, with absent pain sensation distal to the constriction.

Patient presented to us at age of 9-year-old with residual amniotic band, with an angiogram showing a dominant peroneal vessel supplying the right leg that gives off a branch to supply the anterior tibial artery distal to the constriction.

He went through a sequential release of constriction band and soft tissue coverage using an anterolateral thigh fasciocutaneous flap harvested from the contralateral leg. No nerve repair was performed A secondary procedure was performed after 17 days to refashion the flap edge with additional multiple Z plasties.

Two years after the procedure, the sensation improved and the posterior tibial artery became the dominant artery supplying the foot, passing through the previous constriction band.

## Introduction

Amniotic band syndrome consists of congenital anomalies characterized by constriction rings around the limbs, often resulting in deformities and functional impairments. The complex vascular anatomy in deep amniotic band makes free flap transfer a challenging choice for tissue coverage. Deliberation about sensory recovery and vascular changes in relation with amniotic band syndrome is limited in the literature. We report this patient to highlight the improvement of nerve and vascularity of the leg after constriction release and coverage with fasciocutaneous flap.

## Case presentation, surgical procedure, and outcomes

A 9-year-old boy, with a history of previous constriction band release at the age of three, presented with limping. He had sensory loss and occasional wounds on the sole of right foot. Otherwise he was able to walk and run .

**Figure 1:**
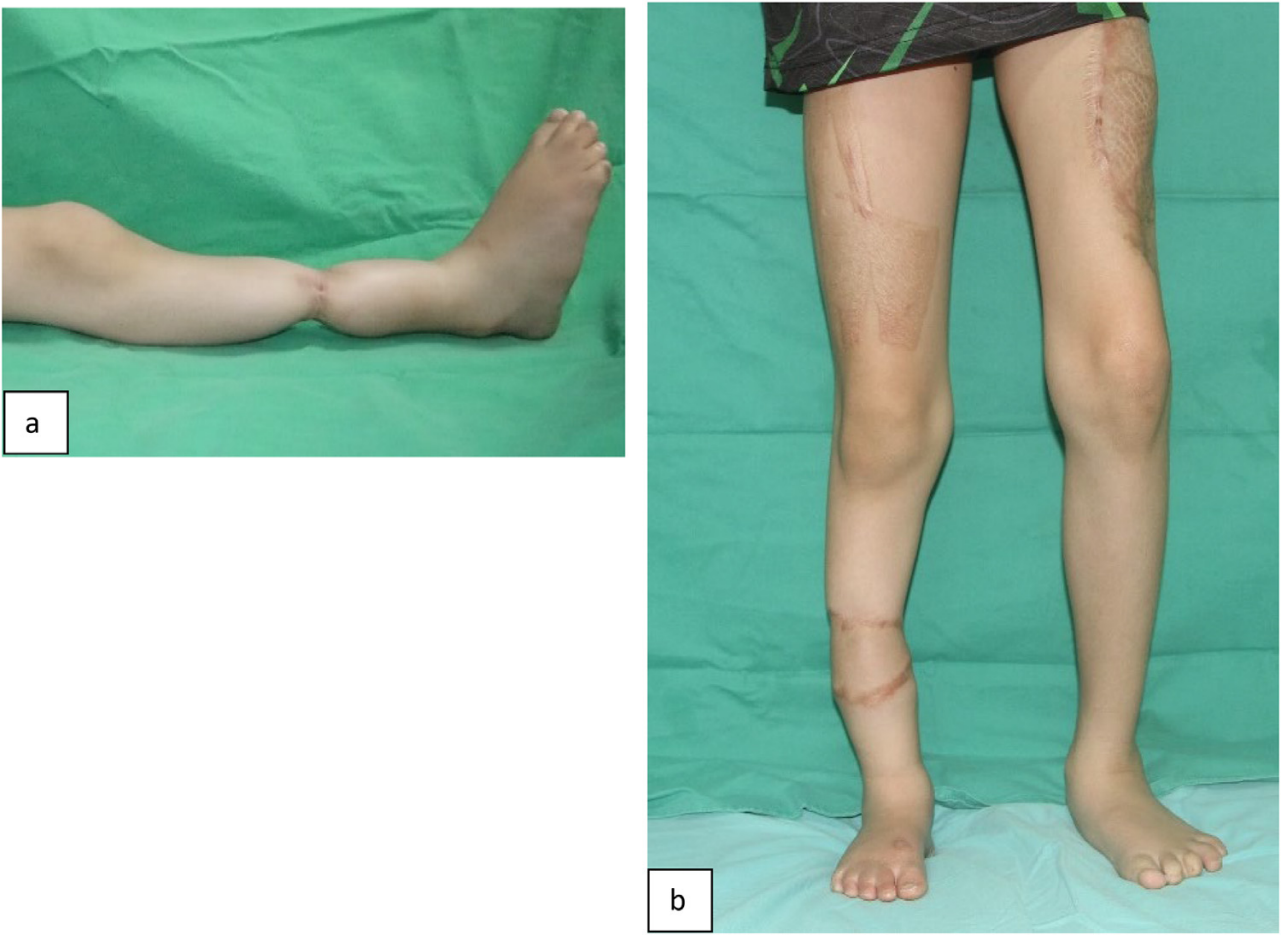
(a) Right leg showing remaining constriction band at the age of seven. (b) 2 years after surgery, child is standing with improved right leg contour and acceptable scars.

His right leg had a well-healed surgical scar at mid-leg without distal edema (Figure 1(a)). Multiple callosities were seen over the sole of his right foot. The right tibia was angulated and shorter by 3 cm. The dorsalis pedis and posterior tibial arteries were vaguely palpable. Sensory loss of sharp pain was noted distal to the constriction band, but vibration and proprioception remained intact.

**Figure 2:**
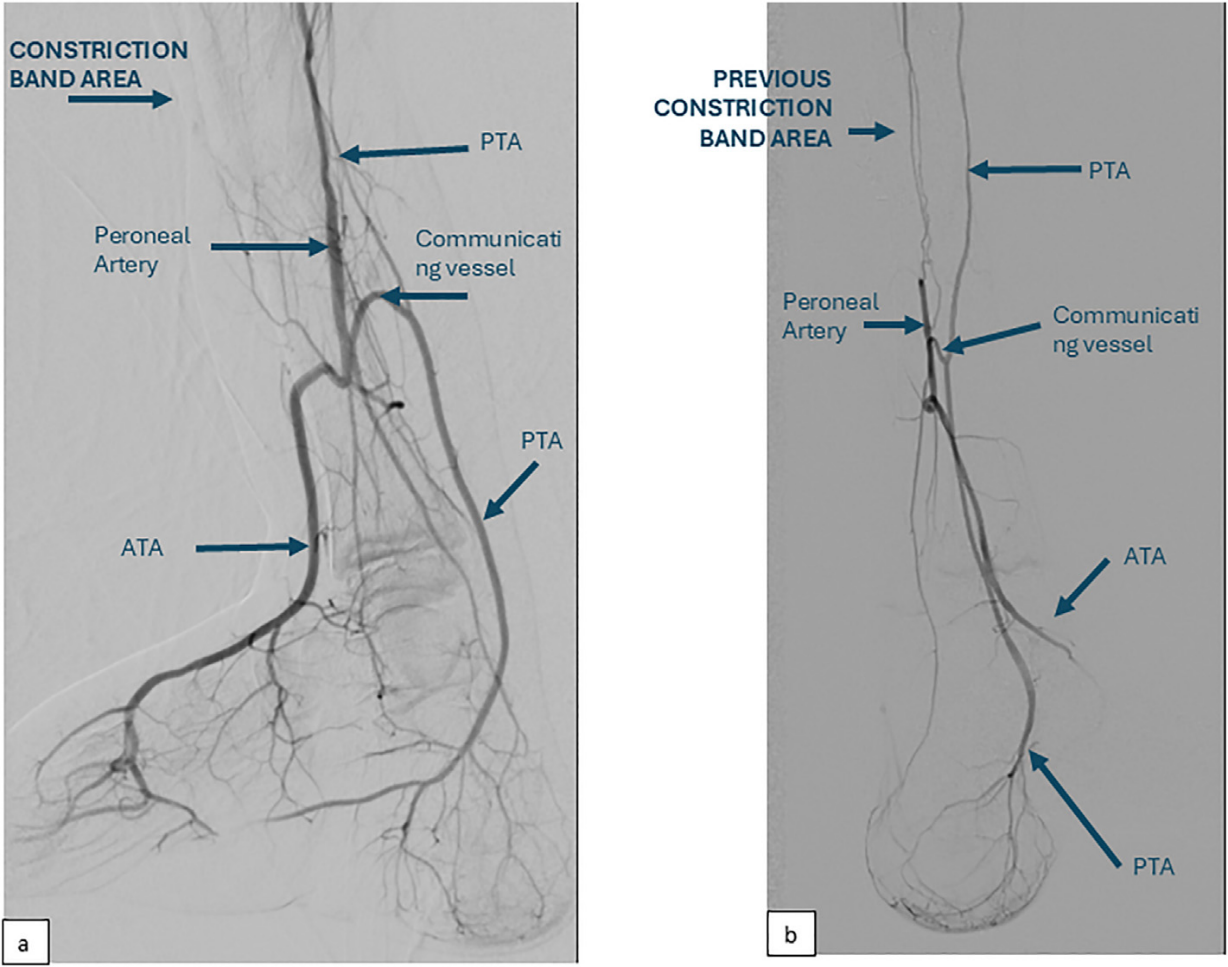
(a) Preoperative angiogram demonstrates the dominance of the peroneal artery in supplying the distal foot reconstituting distal ATA. Posterior tibial artery (PTA) appear to be of smaller caliber. ATA emerges from Popliteal Artery eventually peter out at the area of constriction band. A communication branch is seen between peroneal artery and PTA that eventually supply distal ATA. (b) Two years post constriction band release, PTA appear to increase in caliber, and supply peroneal artery and ATA via the communicating branch distal to the previously constricted band.

Angiogram showed normal arteries until the popliteal trifurcation. The anterior tibial artery (ATA) gradually petered out before reaching the constriction zone (Figure 2a). At the mid-shin level, the posterior tibial artery (PTA) appeared to be of small caliber, while the peroneal artery had a bigger caliber and served as the dominant blood supply to the foot. A communicating branch was observed between the peroneal artery and the PTA distal to the constriction band. The peroneal artery also gave off a branch to reconstitute the distal ATA that continues as the dorsalis pedis artery.

The patient underwent release and excision of the right leg constricting band. An anterolateral thigh (ALT) fasciocutaneous flap was harvested from the contralateral side. Following the release of the constriction band on the right leg, the posterior branch of the peroneal artery, and a tributary of the great saphenous vein proximal to the constriction band were identified and anastomosed with the vessels of ALT fasciocutaneous flap. No nerve repair was performed. Seventeen days post-surgery, the flap edges were refashioned with multiple Z-plasties (Figure 1b). The postoperative recovery was uneventful.

Two years postoperatively, the sensory distal to the constriction level revealed static two-point discrimination of 2–3 mm. The medial plantar, medial calcaneal, and lateral plantar branch areas showed static two-point discrimination of 7 mm, and moving two-point discrimination of 3–5 mm. Semmes-Weinstein monofilament testing of the affected foot indicated sensation thresholds of 5.88–6.10 g at the big toe and metatarsal region, while no sensation was detected in the third toe, little toe, or heel. Sensory recovery was graded as S4, indicating a complete sensory nerve recovery based on the Mackinnon and Dellon grading system. There was no change in active plantar flexion at the ankle.

Repeated angiogram after 2 years demonstrates increased PTA caliber that serves as the dominant vessel of the right leg (Figure 2b). The previously dominant peroneal artery has reduced in caliber. The PTA supplies the distal peroneal artery through retrograde flow, with subsequent perfusion of both the distal peroneal artery and the ATA, establishing it as the primary vascular supply to the foot. Blood supply to the flap is seen with venous drainage to the great saphenous vein.

## Discussion

Neurological deficits secondary to amniotic band syndrome are uncommon, and they more commonly involve sensory deficits rather than motor impairments.^1^ Nerve recovery post-constriction band release varies from case to case, with outcomes depending on multiple factors, including patient age, surgical timing, and intervention type. Weeks^2^ reported a constriction band release alone in the arm of a 3-week-old infant was not enough to improve the major nerve function. The median and ulnar nerves function only improved after exploration and decompression from the deep scar tissue at age of 11 months.^2^ Thus, he suggested that the nerve should be explored.

Sensory recovery in our 9-year-old patient without nerve exploration, who is the oldest among the existing literature, was owing to the radical constriction band release that was permitted by closure with the ATL flap. In addition, the flap provides soft tissue cover with good blood supply to the area for nerve growth and wellbeing. The presence of proprioception and vibration sense prior to release in this patient may indicate a less severe compression of the nerve. We did not attempt to neurotize the flap to avoid disturbing the potential feeder nerve that may compromise its distal tributaries’ improvement after release. Furthermore, the ALT flap at this level is not in contact with any pressure during walking.

In contrast, Uchida and Sugioka (1991) reported no meaningful sensory recovery in three children aged 22 months to 5 years, regardless of surgical intervention. The authors proposed the development of constriction bands after neural decompression, a severe degree of nerve damage, and delayed surgical intervention as the possible reasons.^3^ However, another study on single-stage circumferential release and direct closure had shown motor recovery, in the presence of preserved muscle architecture, in three patients aged between 2 and 22 months.^4^ However the study did not report on sensory change. The ability to flex ankle was not improved owing to the gastrocnemius-soleus structural defect.

Children with deep amniotic bands frequently exhibit vascular anomalies such as absent vessels or incomplete trifurcations.^5^ Preoperative angiography is therefore critical in planning for free tissue transfers. In this case an ALT flap was used for durable coverage, followed by multiple Z-plasties to reduce scar formation and prevent recurrent constriction.

Peroneal artery dominance prior to release can be explained by its protected anatomical courses along the fibula and interosseous membrane, in contrasts to PTA which occupies a more superficial and vulnerable course to constriction. The communicating vessel between peroneal artery and PTA allow for angiosome based vascular adaptation following surgical intervention.

Reestablished PTA dominance and regression of peroneal artery caliber, along with increased PTA-derived perfusion to both the peroneal and ATA distal to the released constriction band are the result of two synergistic mechanisms. First, band release eliminated extrinsic compression on the PTA. Second, the surgical division of the posterior branch of the peroneal artery during flap anastomosis diverted partial flow to the ALT flap while simultaneously reducing native peroneal system perfusion pressure.

A good soft tissue coverage provided by the ALT flap will give a chance for leg lengthening procedure in the future.

## Conclusion

This case highlights the sensory and vascular improvement following the adequate release of the deep constriction band, which was enhanced with vascularized soft tissue cover in a 9-year-old child.

## Patient consent

Parents’ consent was obtained before this report. The consent form states that the parents have given consent for the images and clinical information to be disclosed.

## Funding

None.

## Declaration of competing interest

None declared.

## References

[1] Norman Weinzweig M.D., Arlene Baa M.D. (1994). Radial, ulnar, and median nerve palsies caused by a congenital constriction band of the arm: single-stage correction. Plast Reconstr Surg.

[2] Paul M., Weeks M.D. (1982). Radial, median, and ulnar nerve dysfunction associated with a congenital constricting band of the arm. Plast Reconstr Surg.

[3] Uchida Y., Sugioka Y. (1991). Peripheral nerve palsy associated with congenital constriction band syndrome. J Hand Surg Br.

[4] Dufournier B., Guero S., de Tienda M. (2020). One-stage circumferential limb ring constriction release and direct circular skin closure in amniotic band syndrome: a 14-case series. Orthop Traumatol Surg Res.

[5] Daya M., Makakole M. (2011). Congenital vascular anomalies in amniotic band syndrome of the limbs. J Pediatr Surg.

